# Antibiofilm activity of manogepix, ibrexafungerp, amphotericin B, rezafungin, and caspofungin against *Candida* spp. biofilms of reference and clinical strains

**DOI:** 10.1128/aac.00137-25

**Published:** 2025-05-15

**Authors:** Andres Ceballos-Garzon, Julien Lebrat, Marion Holzapfel, Diego F. Josa, Jeremy Welsch, Derry Mercer

**Affiliations:** 1 BIOASTER Research Institute 397933 , Lyon, France; 2 Studies in Translational Microbiology and Emerging Diseases (MICROS) Research Group, School of Medicine and Health Sciences, Universidad del Rosario 469468 , Bogotá, Colombia; 3 Department of Infectious Diseases, Universidad de La Sabana, Bogota, Colombia; University Children's Hospital Münster, Münster, Germany

**Keywords:** *Candida *biofilms, antibiofilm activity, novel antifungals, Calgary biofilm device

## Abstract

This study comprehensively assessed the activity of manogepix (MNGX), ibrexafungerp (IBF), amphotericin B (AMB), rezafungin (RZF), and caspofungin (CAS) against planktonic cells and mature biofilms of *Candida* spp.—reference and clinical strains using the Calgary biofilm device. Mature-phase biofilms of *C. albicans*, *C. auris (clades I, II, III, IV*), and *C. parapsilosis* were exposed to a range of drug concentrations (0.12–128 µg/mL). Minimum Inhibitory Concentration (MIC) values for planktonic cells were ≤2 µg/mL for all strains; however, biofilm-associated MICs, minimum biocidal concentration (MBC), minimum biofilm eradication (MBEC), and minimum biofilm damaging concentration (MBDC) were significantly higher (2–4,119 times). Geometric mean (GM) of MBEC values indicated that MNGX had the highest antifungal activity within *Candida* species, with a GM-MBEC of 5.9 µg/mL. Despite its overall potency, MNGX was less effective against *C. auris* biofilms from clade IV strains, where IBF showed superior activity. While not the most potent agent overall, AMB induced the smallest fold-change increases (2- to 32-fold) in biofilm-associated states data compared to planktonic MICs. Conversely, CAS exhibited the lowest activity against *Candida* spp. biofilms. The eradication of *C. auris* and *C. parapsilosis* biofilms required substantially higher concentrations than *C. albicans*, with some agents, such as RZF and CAS, necessitating up to 42-fold increases in dosage. In conclusion, our *in vitro* model highlights the antibiofilm activity of novel antifungals against major *Candida* species, revealing significant differences in efficacy among species. MNGX demonstrated the highest activity, underscoring its potential as a promising candidate for the treatment of biofilm-related infections.

## INTRODUCTION

Fungal biofilms are complex communities of microbial cells that attach to abiotic or biotic surfaces and are encased in a self-produced polymeric extracellular matrix (ECM). The ECM is primarily composed of polysaccharides, proteins, nucleic acids, and lipids, which collectively provide mechanical stability, mediate adhesion to surfaces, and form a cohesive, three-dimensional polymer network that interconnects and immobilizes biofilm cells (1). Unlike their planktonic counterparts, biofilm-associated cells adopt a sessile lifestyle, which confers emergent properties such as increased resistance to antimicrobial agents (degree varies by species and antifungal with biofilms withstanding up to 1,000× higher concentrations of antifungals compared to planktonic cells), protection against host immune defenses, and enhanced survival in hostile environments (2). These characteristics significantly complicate the treatment of biofilm-associated infections, often resulting in persistent or recurrent diseases that are particularly challenging to manage (3–5). One critical example is *Candida* spp. bloodstream infection associated with catheter use, which is a major cause of morbidity and mortality, particularly in patients with prolonged intensive care unit (ICU) stays (6, 7).

Among fungal pathogens able to form biofilms, *Candida* spp. are of critical concern (4). Their pathogenicity is compounded by its ability to establish biofilms on a wide range of surfaces, including medical devices such as urinary and vascular catheters, prosthetic joints, and cardiac valves. Planktonic cells released from biofilms may temporarily respond to treatment; however, the biofilm itself typically remains intact, necessitating device removal to achieve resolution (8, 9).

The structural and biochemical diversity of biofilms among *Candida* spp. further exacerbates the difficulty of treating biofilm-related infections (10). For instance, mature biofilms formed by *C. albicans* (the most extensively studied species) are highly heterogeneous, incorporating yeast, hyphal, and pseudohyphal elements, all embedded within the ECM (11). In contrast, *C. glabrata* (*Nakaseomyces glabratus*) biofilms lack hyphal forms and consist of compact layers of yeast cells with an ECM of varying complexity. *C. tropicalis* biofilms exhibit extensive hyphal formation and budding, while *C. parapsilosis* biofilms are characterized by tightly packed yeast clusters and pseudohyphae with minimal ECM (compared to other species, the amount of ECM in *C. parapsilosis* biofilm is low and contains mainly carbohydrates with low protein content) (12). *C. auris (Candidozyma auris*) presents unique challenges in biofilm formation (*C. auris* does not form hyphae and pseudohyphae, but under certain special cultivation conditions, it is able to grow into a pseudohyphae-like form), but the detailed characterization of its biofilm structure and ECM composition remains an active area of research (13). Dominguez et al. previously described that the ECM of *C. auris* is rich in mannan-glucan polysaccharides and demonstrated that their hydrolysis reduces drug tolerance similar to other *Candida* spp. (14). A comprehensive review of biofilm features of *Candida* spp. was recently published (see reference 12).

**Fig 1:**
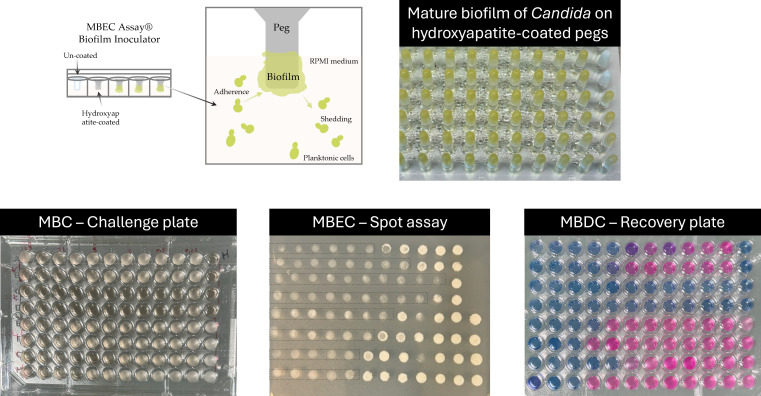
Representative example of *Candida* biofilm formation on the Calgary Biofilm Device and plates used for determining MBC, MBEC, and MBDC. At the top, a schematic representation of biofilm formation on the MBEC Assay device is shown. Biofilm formation is observed as a yellow layer on each peg, while white pegs correspond to negative controls. At the bottom, in the challenge plate, released biofilm cells appear turbid in wells with growth, whereas translucent wells indicate no growth. The spot assay shows fungal growth on Sabouraud Dextrose Agar (SDA) after 24 hours of inoculation (5 µL from each well of the recovery plate). Within the black rectangles, hydroxyapatite powder is visible in the agar; this should not be confused with yeast growth. Finally, the MBDC recovery plate with Alamar Blue reveals metabolic activity, with blue indicating metabolic inactivity and pink indicating metabolic activity.

Considering the importance and the threat that biofilm formation represents, the development of drugs capable of penetrating biofilm structures and overcoming intrinsic tolerance mechanisms is needed. Among the emerging therapies, rezafungin (RZF), ibrexafungerp (IBF), and manogepix (MGPX) have shown promise against a range of fungal pathogens including some that are often resistant to currently available classes of antifungal treatments (15). However, their efficacy against *Candida* spp. biofilms remains poorly explored (16–19). To address this gap, the Calgary biofilm device (CBD) offers a robust and reproducible platform for evaluating biofilm formation and antifungal susceptibility (20). It consists of two parts: a top lid with 96 pegs (coated or uncoated) that form a transferable solid phase and a bottom standard 96-well microtiter plate. The pegs of the lid are designed to fit into the wells of the plate, enabling the growth of 96 individual biofilms on the pegs (Fig. 1). This commercially available *in vitro* system, known as the MBEC Assay System (Innovotech, Canada), is widely used for biofilm-related studies (20, 21). It facilitates experiments assessing biofilm susceptibility and determining the minimum biofilm eradication concentration (MBEC) following exposure to antibiotics and toxic chemicals (22, 23). In this study, we utilize the CBD device for the first time to compare the anti-biofilm activity of RZF, IBF, MGPX, amphotericin B (AMB), and caspofungin (CAS) against mature *C. albicans, C. auris,* and *C. parapsilosis* biofilms.

## RESULTS

The formation of biofilms by *Candida* species was evaluated using hydroxyapatite-coated and uncoated pegs in the Calgary device. The yeasts were able to form biofilms within 24 h only on the hydroxyapatite-coated plates. Consequently, all biofilm-related data were obtained from the hydroxyapatite-coated plates. After 24 h of incubation, no noticeable/significant differences in biofilm formation capacity were observed among the strains (well-documented biofilm producers) (Fig. S1).

The MIC values for planktonic cells, as well as the MBC, MBEC, and MBDC values for each species and antifungal tested, are presented in Table 1. For all *Candida* spp. strains tested, MIC values for AMB, CAS, IBF, MGPX, and RZF were equal to or lower than 2 µg/mL. Differences in the activity of these molecules were observed. Consistently, RZF and MNGX exhibited the highest activity against the different strains of *C. albicans*. A similar pattern was observed for the reference strains of *C. auris* regardless of the clade. However, in the clinical strains of *C. auris* and all *C. parapsilosis* strains, MNGX and IBF were the most effective. Conversely, the antifungals with the lowest activity (i.e., highest MICs) were AMB for *C. albicans* and *C. auris*, and RZF for *C. parapsilosis*.

**TABLE 1:** MIC values for planktonic cells and MBC, MBEC, and MBDC values for biofilm-associated states across *Candida* species and antifungals^*a*^

		(μg/ml)					
Strain	Drug	MIC	MBC	MBC_50_	MBEC	MBEC_50_	MBDC
C. albicans ATCC 90028	RZF	<0.008	16	0.5	0.25	0.25	0.12
	MNGX	0.008	<0.12	<0.12	0.25	0.12	0.12
	IBF	0.03	32	8	1	0.5	0.5
	AMB	0.5	16	4	32	2	8
	CAS	<0.016	>128	2	32	16	64
C. albicans SC5314	RZF	<0.008	<0.12	<0.12	4	2	0.25
	MNGX	<0.008	<0.12	<0.12	1	0.25	0.25
	IBF	0.03	<0.12	<0.12	4	2	32
	AMB	0.5	1	0.5	8	4	8
	CAS	0.016	0.5	0.25	16	8	1
C. albicans ATCC 10231	RZF	<0.008	>128	2	32	1	1
	MNGX	<0.008	0.5	0.12	1	0.25	<0.12
	IBF	0.03	128	0.25	8	0.12	<0.12
	AMB	0.25	8	2	8	0.25	0.12
	CAS	<0.016	64	8	1	0.25	0.5
C. auris (I) DSM 105992	RZF	0.016	>128	2	2	1	2
	MNGX	0.008	<0.12	<0.12	0.5	0.12	0.12
	IBF	0.12	32	8	2	0.5	0.12
	AMB	0.5	4	2	4	4	1
	CAS	0.5	>128	8	8	4	32
C. auris (II) DSM 21092	RZF	0.016	>128	2	2	1	2
	MNGX	0.008	<0.12	<0.12	0.5	0.12	0.12
	IBF	0.12	32	8	2	0.5	0.12
	AMB	1	4	2	4	4	1
	CAS	0.5	>128	8	8	4	32
C. auris (III) DSM 105988	RZF	0.03	>128	>128	>128	1	128
	MNGX	0.016	64	0.5	16	0.5	128
	IBF	0.25	128	64	32	2	32
	AMB	0.5	32	16	64	8	32
	CAS	1	>128	>128	>128	8	128
C. auris (IV) DSM 105990	RZF	0.06	>128	2	64	8	128
	MNGX	0.016	>128	>128	>128	32	128
	IBF	0.25	128	32	16	2	4
	AMB	1	64	32	4	2	2
	CAS	0.5	>128	>128	>128	16	128
C. auris A (IV)	RZF	0.5	>128	128	>128	32	16
	MNGX	0.016	>128	0.12	>128	0.5	32
	IBF	0.03	64	16	64	0.5	32
	AMB	2	16	8	64	16	16
	CAS	0.12	>128	>128	>128	1	32
C. auris C (IV)	RZF	0.25	>128	>128	64	1	16
	MNGX	0.016	128	0.12	64	0.5	0.5
	IBF	0.03	32	8	32	32	0.5
	AMB	1	16	4	32	16	2
	CAS	0.25	>128	>128	>128	>128	32
C. parapsilosis ATCC 22019	RZF	0.5	>128	16	>128	>128	>128
	MNGX	0.008	<0.12	<0.12	16	4	4
	IBF	0.12	64	32	8	2	1
	AMB	1	8	4	16	2	1
	CAS	0.5	>128	>128	>128	>128	>128
C. parapsilosis B	RZF	1	>128	16	16	1	1
	MNGX	0.008	1	0.12	0.25	0.12	0.25
	IBF	0.016	32	16	0.5	0.5	0.5
	AMB	0.12	8	2	0.5	0.5	0.5
	CAS	0.25	128	16	>128	2	2
C. parapsilosis H	RZF	1	128	32	>128	32	64
	MNGX	0.06	0.5	0.25	8	2	0.25
	IBF	0.12	64	32	32	8	64
	AMB	0.5	8	4	8	8	8
	CAS	0.5	64	16	>128	32	16
C. parapsilosis k	RZF	1	>128	64	>128	64	64
	MNGX	0.008	2	0.5	2	0.5	0.5
	IBF	0.016	64	32	1	0.5	0.5
	AMB	1	32	64	1	0.5	1
	CAS	1	128	32	>128	2	32

^*a*^ Within the brackets, the clade is depicted.

Considering the activity of antifungals in eradicating mature *Candida* spp. biofilms, MNGX generally demonstrates the highest antibiofilm activity, being the most potent antifungal in 8 out of 13 strains. Interestingly, in the biofilm of the reference strain *C. auris* DSM 105990 (belonging to clade IV, South America), as well as in the biofilms of the Colombian *C. auris* clinical strains, MNGX was not the most powerful agent (MBEC, ≥64 µg/mL).

When geometric mean (GM) values were considered, MNGX exhibited the highest antifungal activity across all three *Candida* species, with a GM MBEC value of 5.9 µg/mL. Similarly, MNGX demonstrated superior antibiofilm activity, with the lowest MBC, MBEC, and MBDC values for both *C. albicans* and *C. parapsilosis*. However, for *C. auris*, the lowest MBC value was observed with AMB, while the lowest MBEC and MBDC values were achieved with IBF (Table 2).

**TABLE 2:** Geometric mean (GM) values of MIC, MBC, MBEC, and MBDC and fold changes of MBC, MBEC, and MBDC relative to MIC for each antifungal and *Candida* species^*a*^

Specie	Drug	GM	GM	GM	GM	MBC	MBEC Fold change	MBDC Fold change
		MIC	MBC	MBEC	MBDC	Fold change		
*C. albicans*	RZF	0.01	7.89	3.17	0.31	**789**	**317**	31
*C. auris*		0.06	256	32	16	4,119	**515**	257
*C. parapsilosis*		0.84	215	128	32	256	152	38.1
*C. albicans*	MNGX	0.01	0.19	0.63	0.15	19	63	15
*C. auris*		0.01	14.1	14.3	3.9	**1,107**	**1,122**	311
*C. parapsilosis*		0.01	0.59	2.83	0.59	44.5	214	44.9
*C. albicans*	IBF	0.03	7.89	3.17	1.24	263	106	41.4
*C. auris*		0.1	57	12.7	1.76	**591**	132	18.2
*C. parapsilosis*		0.04	53.8	3.36	2	**1,228**	76.8	45.6
*C. albicans*	AMB	0.4	5.04	12.7	1.97	12.7	32	4.97
*C. auris*		0.89	13.9	14.3	3.56	15.6	16	4
*C. parapsilosis*		0.49	11.3	2.83	1.41	22.9	5.71	2.86
*C. albicans*	CAS	0.02	20.16	8	3.17	**1,260**	**500**	198
*C. auris*		0.39	256	80.6	50.8	**649**	205	129
*C. parapsilosis*		0.5	128	256	22.6	256	512	45.3

^*a*^ GM: geometric mean. MIC: Minimum Inhibitory Concentration, the lowest antifungal concentration that inhibits visible growth of planktonic cells (50% for RZF, MNGX, IBF, CAS). MBC: Minimum Biocidal Concentration, the lowest concentration of an antifungal agent that eradicates ≥99% of the population of both planktonic-embedded cells and dispersed cells shed from the biofilm. MBEC: Minimum Biofilm Eradication Concentration, the lowest antifungal concentration needed to eradicate pre-formed biofilms. MBDC: Minimum Biofilm Damaging Concentration, the antifungal concentration that inhibits the metabolic activity of biofilm-associated cells. The bolded fold change values (MBC, MBEC, and MBDC relative to MIC) represent the highest fold changes.

Conversely, under the MBC criterion, CAS appears to be the least effective antifungal against *C. albicans* and *C. auris* cells shed from the biofilm, while RZF is the least effective against *C. parapsilosis*. Regarding the MBEC, CAS appears to be the least effective against mature *C. auris* and *C. parapsilosis* biofilms, while AMB is the least effective against *C. albicans* biofilms. For the MBDC, CAS is the least effective at inhibiting the metabolic activity of *Candida* spp. biofilms for *C. albicans* and *C. parapsilosis*, while RZF is the least effective against *C. parapsilosis*. In summary, MNGX demonstrated the highest activity among the five molecules evaluated, whereas CAS was the least potent molecule against *Candida* spp. biofilms.

**Fig 2:**
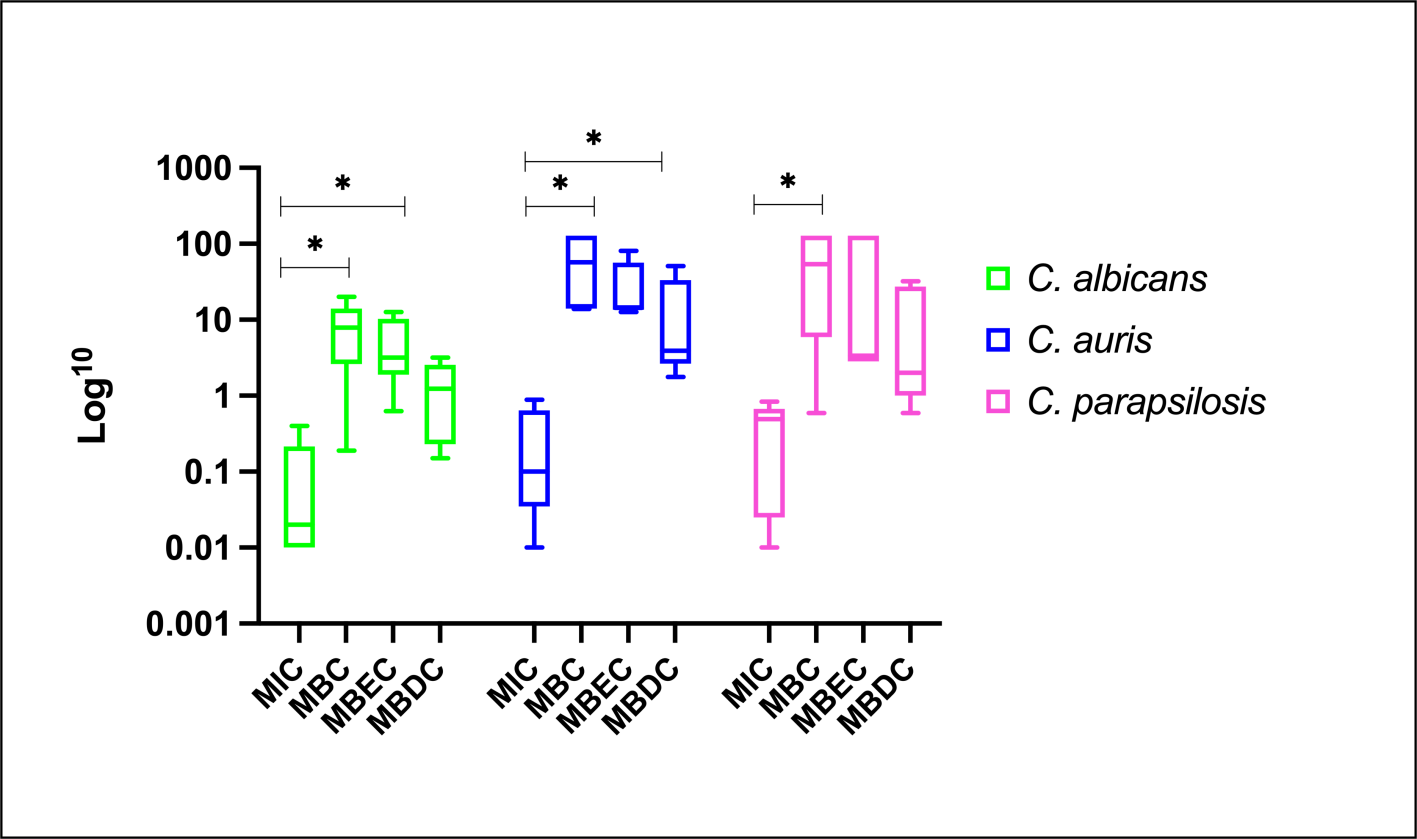
Overall effect of antifungals on planktonic and biofilm-associated cells: base-10 logarithm transformed geometric means of MIC, MBC, MBEC, and MBDC for each *Candida* species.

Although MNGX was the most active agent, AMB induced the smallest fold-change increase (2- to 32-fold) when comparing MBC, MBEC, and MBDC to MIC. Conversely, the largest increases were observed with CAS in *C. albicans*; with RZF (MBC) and MNGX (MBEC and MBDC) in *C. auris*; and with IBF (MBC, MBDC) and MNGX (MBEC) in *C. parapsilosis* (Table 2). As expected, the highest values were observed when MBC was compared to the MICs of planktonic cells (MBCs are generally higher than MICs because they assess cells that, although planktonic, have a higher level of tolerance to the antifungal due to their prior association with a biofilm and the use of experimental conditions with higher inoculum), followed by MBEC and MBDC values, regardless of the species or antifungal agent. Similar patterns were observed for all three species. The MBC and MBEC values were relatively close to each other, unlike the MBDC, which were lower. Since the MBDC reflects a metabolic observation taken over a short period, these results are consistent and expected (Fig. 2).

The statistical analysis revealed notable differences when the MIC values were compared with the concentrations obtained for MBC, MBEC, and MBDC (representing biofilm-associated states) for different *Candida* species and antifungal agents (Table 3). Statistical differences reflect the increased difficulty of eradicating/inhibiting biofilms, evidenced by reduced antifungal activity (fold-change) changes compared to planktonic cells (MIC). For *C. albicans*, CAS showed a statistically significant difference between MIC and MBC (*P* = 0.0238), reflecting a marked increase in the concentration required to eradicate cells shed from the biofilm compared to planktonic cells.

**TABLE 3:** Shifts in antifungal activity between planktonic and biofilm forms of *Candida^a^*

Specie	Drug	Comparison	*P* value		Comparison	*P* value	
*C. albicans*	CAS	MIC vs MBC	0.0238	*			
*C. auris*	RZF	MIC vs MBC	<0.0001	****	MBC vs MBEC	0.0003	***
		MIC vs MBEC	0.0084	**	MBC vs MBDC	<0.0001	****
	MNGX	MIC vs MBC	0.0034	**			
		MIC vs MBEC	0.0166	*			
	CAS	MIC vs MBC	<0.0001	****	MBC vs MBDC	<0.0001	****
		MIC vs MBEC	<0.0001	****	MBEC vs MBDC	0.013	*
*C. parapsilosis*	RZF	MIC vs MBC	<0.0001	****	MBC vs MBDC	0.0048	**
		MIC vs MBEC	<0.0001	****	MBEC vs MBDC	0.0403	*
		MIC vs MBDC	0.0306	*			
	CAS	MIC vs MBC	0.0007	***	MBC vs MBEC	0.0167	*
		MIC vs MBEC	<0.0001	****	MBEC vs MBDC	<0.0001	****

^*a*^ Two-way analysis of variance (ANOVA) and post-hoc comparisons with Dunnett’s multiple comparison tests [*P* < 0.03 (*); *P* < 0.0021 (**); *P* < 0.0002 (***); *P* < 0.0001 (****)]. Observed statistical differences among MIC, MBC, MBEC, and MBDC are attributed to greater shifts in antifungal activity displayed by certain antifungals when transitioning from planktonic cells to biofilms.

For *C. auris*, RZF demonstrated highly significant differences between MIC and MBC (*P* < 0.0001) and between MIC and MBEC (*P* = 0.0084), indicating a substantial increase in concentration required to target biofilm-related states. Similarly, MNGX exhibited significant differences between MIC and MBC (*P* = 0.0034) and MIC and MBEC (*P* = 0.0166). CAS displayed the largest fold changes, with highly significant differences observed between MIC and both MBC and MBEC (*P* < 0.0001 for both), highlighting its reduced efficacy against *C. auris* biofilms.

For *C. parapsilosis*, RZF showed highly significant differences for MIC vs MBC and MIC vs MBEC (*P* < 0.0001 for both), and a significant difference for MIC vs MBDC (*P* = 0.0306). CAS also demonstrated notable differences, with a significant increase for MIC vs MBC (*P* = 0.0007) and a highly significant increase for MIC vs MBEC (*P* < 0.0001).

When comparing MBC, MBEC, and MBDC, significant differences emerged, particularly for the echinocandin drugs RZF and CAS. For *C. auris*, RZF exhibited significant differences between MBC and MBEC values (*P* = 0.0003) and between MBC and MBDC (*P* < 0.0001). This indicates a substantial increase in the concentration needed to eliminate mature biofilms and reduce metabolic activity. CAS displayed highly significant differences between MBC vs MBEC and MBC vs MBDC (*P* < 0.0001 for both), along with a significant difference between MBEC vs MBDC (*P* = 0.013), suggesting a consistent escalation in required concentrations across biofilm states.

In *C. parapsilosis*, RZF showed significant differences between MBC vs MBEC (*P* = 0.0048) and MBEC vs MBDC (*P* = 0.0403), while CAS demonstrated significant differences between MBC vs MBEC (*P* = 0.0167) and highly significant differences between MBEC vs MBDC (*P* < 0.0001). These findings indicate that the transition from eradicating cells shed from biofilms to targeting mature biofilms and inhibiting metabolic activity requires significantly different concentrations of echinocandin drugs.

## DISCUSSION

Biofilms represent a significant challenge in both clinical and environmental settings due to their inherent resilience and ability to tolerate hostile conditions (24, 25). From an environmental perspective, biofilms contribute to microbial persistence on various surfaces, playing a role in contamination and the spread of pathogens (6, 26). Therefore, the development and evaluation of molecules with significant antibiofilm activity is crucial.

In this study, AMB, CAS, IBF, MGPX, and RZF demonstrated good activity against the planktonic cells of *Candida* spp. However, all strains showed higher biofilm-associated MICs (MBC, MBEC, and MBDC) for nearly all five agents, with values ranging from 2 to 4,119 times greater than those observed for planktonic cells. Among the tested antifungals, MNGX displayed the highest anti-biofilm activity, positioning it as the most potent agent of those tested.

MNGX, the active moiety of the prodrug fosmanogepix (formerly APX001), has emerged as a promising drug due to its broad-spectrum activity and unique mechanism of action targeting the fungal Gwt1 enzyme. By inhibiting Gwt1, MNGX blocks inositol acylation during the synthesis of glycosylphosphatidylinositol (GPI)-anchored fungal cell wall proteins, which are crucial for maintaining cell wall integrity and key fungal processes (e.g., adhesins, whose role in cell-cell adhesion and biofilm formation is well established) (15, 18, 27). By disrupting GPI anchor biosynthesis, MNGX prevents the proper localisation and function of cell surface proteins essential for biofilm establishment. Specifically, in *C. albicans*, MNGX inhibits hyphal growth and reduces adherence to host tissues or abiotic surfaces, both of which are pivotal in biofilm development (18). This provides a compelling explanation for the superior activity of MNGX against mature biofilms. Nevertheless, its limited activity against biofilms of *C. auris* strains of the South American clade is noteworthy and cannot be easily explained. The reduced susceptibility of clade IV biofilms to MNGX may be linked to clade-specific genomic variations. Studies comparing global *C. auris* isolates have highlighted clade-specific differences in drug resistance and the underlying mechanisms, including the evolution of cell wall proteins that influence antifungal susceptibility and host interactions (28, 29). Recently, Hirayama et al. demonstrated that *TAC1b* mutations in *C. auris* decrease MNGX susceptibility owing to increased *CDR1* expression (30). These findings suggest that Clade IV strains may possess multiple adaptations that enhance biofilm response to MNGX. However, further research with a broader range of isolates is needed to confirm these observations and clarify the mechanisms driving these clade-specific differences.

AMB, while not the most potent antifungal overall, induced the smallest fold-change increases (2- to 32-fold) when comparing MBC, MBEC, and MBDC to MIC. This observation suggests that AMB’s activity is less impacted by the protective biofilm matrix, consistent with findings by Chatzimoschou et al.*,* who reported that AMB deoxycholate exhibited stable efficacy against mature *C. auris* biofilms despite requiring higher concentrations than in planktonic states (31). Melo et al. reported that *C. albicans*, *C. tropicalis*, *C. orthopsilosis*, and *C. metapsilosis* exhibit higher susceptibility to AMB compared to *C. parapsilosis* (32). However, in our study, AMB effectively eradicated *C. parapsilosis* biofilms at lower doses (MBEC values) than those required for *C. albicans*. Similarly, Kuhn et al*.* observed that AMB was effective against *C. albicans* biofilms at low doses (≤8 µg/mL) (33).

In this study, IBF (SCY-078) demonstrated good efficacy against *Candida* spp. biofilms (GM 3–12 µg/mL), corroborating the findings of Larkin et al.*,* who highlighted its broad-spectrum activity and efficacy against both planktonic and biofilm-associated *C. auris* (4 µg/mL) (34). Similarly, Marcos-Zambrano et al. reported that IBF was active *in vitro* against preformed mature *C. albicans*, *C. parapsilosis*, and *C. tropicalis* biofilms. Notably, *C. glabrata* showed the most attenuated response to IBF in both planktonic and biofilm forms (17).

While echinocandins have previously been shown to exhibit low biofilm MICs, compared to planktonic MICs in *Candida* spp. (19, 33, 35–38), we observed that CAS and RZF displayed significantly higher biofilm-associated MICs across all *Candida* species tested, particularly for CAS. Similarly, a study on *C. auris* reported (MBEC) values ranging from 100 to 3,000 times higher than those for planktonic cells, highlighting substantial biofilm-related resistance (31). Even though resistant strains were not included in this study, other research indicates that the biofilm formation of echinocandin-resistant strains is comparable to that of wild-type strains although resistance to echinocandins remains high. In that study, liposomal AMB demonstrated the highest activity against *fks* mutant *Candida* biofilms (39). Renátó Kovács et al*.* also reported that MCF and CAS were active against *Candida* spp. biofilms *in vitro*, with MICs ranging between 32–256 μg/mL and 16–512 μg/mL, respectively (40). In our study, we observed species-specific differences: for *C. albicans*, the GM MBEC was relatively low (8 µg/mL), whereas it was notably higher for *C. auris* and *C. parapsilosis*, with GMs of 80 µg/mL and 256 µg/mL, respectively. Additionally, Chandra et al*.* found that RZF was effective against *C. albicans* biofilms at concentrations as low as 0.25 µg/mL using a catheter-associated biofilm model [elastomer (SE) catheter discs] (19). For RZF, we also observed similar differences as with CAS, with higher MBECs for *C. auris* and *C. parapsilosis*.

Differences in antifungal activity revealed that eradicating *C. auris* and *C. parapsilosis* biofilms required significantly higher doses of antifungal than *C. albicans*. In some cases, these increases were substantial, with antifungals such as RZF requiring up to 42 times higher doses for *C. parapsilosis* biofilms (GM MBEC of 128 µg/mL) compared to *C. albicans* (GM MBEC of 3 µg/mL). Similarly, CAS showed an increase of 13-fold for *C. auris* (GM MBEC of 80.6 µg/mL) compared to *C. albicans* (GM MBEC of 8.00 µg/mL), and up to 32 times higher for *C. parapsilosis* (GM MBEC of 256 µg/mL). MNGX demonstrated superior activity overall but still required approximately 75 times higher concentrations to eradicate *C. auris* biofilms (GM MBEC of 14.3 µg/mL) compared to *C. albicans* (GM MBEC of 0.19 µg/mL). These findings underscore the significant variation in antifungal efficacy among species and highlight the increased difficulty of eradicating biofilms of *C. auris* and *C. parapsilosis*.

Interestingly, higher MBC values were observed compared to MBEC values. This could be attributed to the tolerance mechanisms and stress responses activated during biofilm formation and antifungal exposure. Throughout the biofilm growth cycle, biofilms continuously release planktonic yeast cells, which are subjected to significant stress, such as nutrient limitation, oxidative stress, and exposure to sublethal antifungal concentrations. This stress triggers adaptive responses, including the upregulation of efflux pumps, cell wall remodelling, and metabolic adaptations, resulting in a heterogeneous population with increased antifungal resistance (41). Additionally, the MBC parameter accounts for the eradication of both planktonic and biofilm-dispersed cells. This heterogeneity, combined with the protective mechanisms of biofilms, likely explains the higher MBC values.

Some limitations should be considered in interpreting these findings. First, the study was limited to a few reference and clinical strains, which may not encompass the full spectrum of variability within *Candida* species. Second, the study focused on single-agent activity, whereas combination therapies are often used for biofilm-associated infections (42). Future studies should explore the synergistic potential of antifungal combinations, both *in vitro* and *in vivo*. On the other hand, the high cost of CBD poses a significant barrier to accessibility, particularly in low- and middle-income countries (LMICs). Therefore, determining the MBC and MBDC represents a valuable alternative for assessing antifungal activity against biofilms.

In summary, our study provides a detailed analysis of the antibiofilm activity of five clinically relevant antifungal agents. MNGX emerged as the most potent antibiofilm antifungal overall although its efficacy varied depending on species and strain. AMB demonstrated consistent activity with minimal fold-change increases from the MIC, while CAS exhibited limited efficacy in some biofilm-associated states. These findings are consistent with and expand upon previous studies, reinforcing the need for optimised antifungal strategies tailored to biofilm-associated infections. Further research is necessary to explore the molecular mechanisms underlying species-specific and strain-specific variations in biofilm susceptibility to antifungal agents, particularly for emerging pathogens like *C. auris*.

## MATERIALS AND METHODS

### Isolates and identification

A total of 13 strains were used, including laboratory reference strains: *C. albicans* ATCC 10231, *C. albicans* ATCC 90028, *C. albicans* SC5314/MYA-2876, *C. auris* DSM 105992 (South Asia—Clade I)/CDC AR-Bank 0387, *C. auris* DSM 21092 (East Asia—Clade II)/CBS 10913, *C. auris* DSM 105988 (South Africa—Clade III)/CDC AR-Bank 0383, *C. auris* DSM 105990 (South America—Clade IV)/CDC AR-Bank 0385, C. *parapsilosis* ATCC 22019/CBS 604, and clinical strains: *C. auris* A (blood), *C. auris* C (blood), *C. parapsilosis* B (blood), *C. parapsilosis* H (bronchoalveolar lavage), and *C. parapsilosis* K (rib cage) were used. All clinical strains were obtained from a health institution in Bogotá, Colombia.

The strain set was preserved at −80°C (glycerol stocks). In the week of the experiments, the strains were inoculated onto Sabouraud Dextrose Agar (SDA) plates and chromogenic media, i.e., CHROMagar Candida Plus (CHROMagar, Paris, France) followed by incubation at 35°C for 24–36 h. The identity of all strains was confirmed using the Vitek MS system (bioMérieux, Inc.), and the analysis was performed according to the manufacturer’s instructions. Briefly, a small portion of a single colony was directly spotted onto a target plate and covered with 0.5 µL formic acid (bioMérieux) and allowed to dry before being loaded into the Vitek MS. All strains were considered correctly identified with an acceptable confidence value of ≥99%.

### Antifungal susceptibility testing

Reference AFST was carried out following the CLSI-M27 4^th^ed (43) guidelines. Antifungal drugs included AMB, CAS, IBF, MGPX, and RZF (0.002% tween 20-supplemented). The antifungal drugs were purchased from Sigma-Aldrich (France) or obtained from their manufacturers as standard powders. Drug-free and yeast-free controls were included, and microtiter plates (Thermo Scientific Nunc 96-Well, Nunclon Delta-Treated, Round Bottom (U) REF 10344311) were incubated at 35°C and read visually after 24 h.

The MIC endpoints for echinocandins, IBF, and MGPX were defined as the lowest drug concentration that caused a prominent decrease in visual growth in comparison with the control-well containing media only. For AMB, the MIC was defined as the lowest concentration at which there was full inhibition of visual growth relative to the drug-free control wells. *C. krusei* ATCC 6258 and *C. parapsilosis* ATCC 22019 strains were used as quality control strains (44). For IBF assays, the strain *C. albicans* ATCC 90028 was used as quality control (45).

### *In vitro* antibiofilm susceptibility test against established biofilms

*Candida* spp. strains were streaked onto SDA plates from glycerol stocks 3–5 days prior to the assay and incubated at 37°C. Colonies were then subcultured onto fresh SDA plates to ensure viability and consistency. To prepare the inoculum, cells from the second streak were harvested and adjusted to a 0.5 McFarland standard (~1 × 10⁶ cells/mL) in RPMI 1640 medium (Sigma-Aldrich) with morpholinepropanesulfonic acid (MOPS, Sigma-Aldrich) and 2% glucose (Sigma-Aldrich). The inoculum was introduced into the 96-well plate of the MBEC Assay Biofilm Inoculator (Innovotech, Canada). Two plates were prepared: one for assessing biofilm formation capacity and the other for evaluating the antibiofilm activity of antifungal agents. A volume of 150 µL was pipetted into each well, including designated negative control wells (A12–D12) and positive control wells (E12–H12). The volume of inoculum was calibrated to ensure uniform biofilm formation on the pegs that would be fully immersed in the subsequent antimicrobial challenge plate. The peg lids, both uncoated and hydroxyapatite-coated (facilitates biofilm growth by fastidious microorganisms, including *Candida* spp.) REF SKU: 19141, were placed onto the microtitre plate bases, ensuring proper alignment (e.g., peg A1 with well A1). The assembled devices were incubated at 37°C in a humidified incubator on a platform shaker set to 110 rpm for 24 h to promote biofilm formation. Following incubation, a rinsed plate was prepared by filling each well of a new sterile 96-well plate with 200 µL of sterile saline. The MBEC Assay lid with biofilm growth was transferred to the rinse plate and gently submerged for 10 s to remove planktonic cells.

To determinate biofilm formation capacity of each strain, the MBEC Assay lid (after rinsing) was transferred to a recovery plate containing 150 µL of the RPMI + MOPS + GLU 2% medium per well and allowed to equilibrate for 30 min. To detach the biofilms, the recovery plate was subjected to sonication for 30 min at high frequency. Following sonication, 10 µL of 700 µM resazurin (Sigma–Aldrich) was added to each well and incubated at 37°C for 4 h. The biofilm was quantified indirectly by measuring the fluorescent water-soluble resorufin product which is generated when resazurin is reduced through metabolic activity associated with respiration. Fluorescence was measured at an excitation wavelength of 560 nm and an emission wavelength of 590 nm. The results were expressed in arbitrary fluorescence units (AU) (46).

For the second plate, after rinsing, the lid was transferred to a challenge plate containing antifungal agents AMB, CAS, IBF, MGPX, and RZF at concentrations ranging from 0.12 to 128 µg/mL in twofold dilutions. The plate was then incubated for an additional 24 h at 37°C with constant agitation.

### Minimum biocidal concentration

The challenge plate was visually inspected to determine the MBC and the MBC₅₀. The MBC represents the lowest concentration of an antifungal agent that eradicates ≥99% of the population of both planktonic-embedded cells and dispersed cells shed from the biofilm, as evidenced by visibly clear wells following the chosen contact time. The MBC₅₀ is defined as the concentration at which yeast growth is reduced by 50% compared to the untreated control based on visual assessment (47).

### Minimum biofilm eradication concentration

The MBEC Assay lid (after a rinsing) was then transferred to a recovery plate containing 150 µL of the RPMI + MOPS + GLU 2% medium per well and allowed to equilibrate for 30 min. To detach the biofilms, the recovery plate was placed in a sonicator for 30 min at high frequency. For this step, the plate was placed on a stainless-steel tray inside the sonicator (water up to half the height of the plate), covered with Parafilm and aluminium foil to avoid contamination. After sonication, biofilm viability was confirmed by plating 5 µL from each well onto SDA plates, which were incubated at 37°C for 24 h. The MBEC was defined as the lowest antifungal concentration at which no growth was observed on agar plates (48). The MBEC_50_ values were calculated by performing colony-forming unit (CFU) counts. The CFU counts were compared to the growth control (biofilm without antifungal treatment), and the MBEC_50_ was defined as the lowest antifungal concentration that reduced biofilm growth by 50% compared to the control.

### Minimum biofilm damaging concentration

To determine the MBDC, 20 µL of AlamarBlue (20×) Reagent (Sigma-Aldrich, France) was added to each well of the recovery plate and incubated for 4 h (with some strains requiring extended incubation). Biofilm metabolic activity was assessed indirectly by measuring fluorescence using a plate reader with excitation/emission settings of 560/590 nm. Metabolically active cells reduce resazurin (blue) to resorufin (pink). The MBDC was defined as the lowest antifungal concentration that caused damage or inhibited the metabolic activity of *Candida* spp. biofilms, resulting in the absence of a color change (blue wells) and no detectable fluorescence signal. Unlike the MIC, which applies to planktonic cells, the MBDC reflects the impact of antifungal agents on cells within a developed biofilm, assuming that most of these cells are already compromised (49, 50).

### Data analysis

All experiments were performed in triplicate. For statistical analysis, high off-scale values were converted to the next highest concentration, and low off-scale results were left unchanged. Two-way analysis of variance (ANOVA) and post-hoc comparisons with Dunnett’s multiple comparison tests were used to evaluate the significance of differences. Statistical significance was defined as [*P < *0.03 (*); *P  *<  0.0021 (**); *P  *<  0.0002 (***)*; P  *<  0.0001 (****)]. Analysis was carried out using the GraphPad Prism software (v10).

## SUPPLEMENTAL MATERIAL

Fig. S1 aac.00137-25-s0001.pdf Biofilm formation capacity of *Candida albicans*, *Candida auris*, and *Candida parapsilosis* assessed using the Calgary Biofilm Device.

ASM does not own the copyrights to Supplemental Material that may be linked to, or accessed through, an article. The authors have granted ASM a non-exclusive, world-wide license to publish the Supplemental Material files. Please contact the corresponding author directly for reuse.

## References

[1] Flemming HC, Wingender J. 2010. The biofilm matrix. Nat Rev Microbiol 8:623–633. doi:10.1038/nrmicro2415 20676145

[2] Taff HT, Mitchell KF, Edward JA, Andes DR. 2013. Mechanisms of Candida biofilm drug resistance. Future Microbiol 8:1325–1337. doi:10.2217/fmb.13.101 24059922 PMC3859465

[3] Wall G, Montelongo-Jauregui D, Vidal Bonifacio B, Lopez-Ribot JL, Uppuluri P. 2019. Candida albicans biofilm growth and dispersal: contributions to pathogenesis. Curr Opin Microbiol 52:1–6. doi:10.1016/j.mib.2019.04.001 31085405 PMC6842673

[4] Ramage G, Martínez JP, López-Ribot JL. 2006. Candida biofilms on implanted biomaterials: a clinically significant problem. FEMS Yeast Res 6:979–986. doi:10.1111/j.1567-1364.2006.00117.x 17042747

[5] Horton MV, Nett JE. 2020. Candida auris infection and biofilm formation: going beyond the surface. Curr Clin Microbiol Rep 7:51–56. doi:10.1007/s40588-020-00143-7 33178552 PMC7654955

[6] Wijaya M, Halleyantoro R, Kalumpiu JF. 2023. Biofilm: the invisible culprit in catheter-induced candidemia. AIMS Microbiol 9:467–485. doi:10.3934/microbiol.2023025 37649801 PMC10462453

[7] Atiencia-Carrera MB, Cabezas-Mera FS, Tejera E, Machado A. 2022. Prevalence of biofilms in Candida spp. bloodstream infections: a meta-analysis. PLoS ONE 17:e0263522. doi:10.1371/journal.pone.0263522 35113972 PMC8812928

[8] Bouza E, Guinea J, Guembe M. 2014. The role of antifungals against Candida biofilm in catheter-related candidemia. Antibiotics (Basel) 4:1–17. doi:10.3390/antibiotics4010001 27025612 PMC4790322

[9] Cornely OA, Sprute R, Bassetti M, Chen SC-A, Groll AH, Kurzai O, Lass-Flörl C, Ostrosky-Zeichner L, Rautemaa-Richardson R, Revathi G, et al.. 2025. Global guideline for the diagnosis and management of candidiasis: an initiative of the ECMM in cooperation with ISHAM and ASM. Lancet Infect Dis:S1473-3099(24)00749-7. doi:10.1016/S1473-3099(24)00749-7 39956121

[10] Cavalheiro M, Teixeira MC. 2018. Candida biofilms: threats, challenges, and promising strategies. Front Med (Lausanne) 5:28. doi:10.3389/fmed.2018.00028 29487851 PMC5816785

[11] Lohse MB, Gulati M, Johnson AD, Nobile CJ. 2018. Development and regulation of single- and multi-species Candida albicans biofilms. Nat Rev Microbiol 16:19–31. doi:10.1038/nrmicro.2017.107 29062072 PMC5726514

[12] Malinovská Z, Čonková E, Váczi P. 2023. Biofilm formation in medically important Candida species. J Fungi (Basel) 9:9. doi:10.3390/jof9100955 PMC10607155 37888211

[13] Wang TW, Sofras D, Montelongo-Jauregui D, Paiva TO, Carolus H, Dufrêne YF, Alfaifi AA, McCracken C, Bruno VM, Van Dijck P, Jabra-Rizk MA. 2024. Functional redundancy in Candida auris cell surface adhesins crucial for cell-cell interaction and aggregation. Nat Commun 15:9212. doi:10.1038/s41467-024-53588-5 39455573 PMC11511831

[14] Dominguez EG, Zarnowski R, Choy HL, Zhao M, Sanchez H, Nett JE, Andes DR. 2019. Conserved role for biofilm matrix polysaccharides in Candida auris drug resistance. mSphere 4:e00680-18. doi:10.1128/mSphereDirect.00680-18 30602527 PMC6315084

[15] Hoenigl M, Sprute R, Egger M, Arastehfar A, Cornely OA, Krause R, Lass-Flörl C, Prattes J, Spec A, Thompson GR 3rd, Wiederhold N, Jenks JD. 2021. The antifungal pipeline: fosmanogepix, ibrexafungerp, olorofim, opelconazole, and rezafungin. Drugs (Abingdon Engl) 81:1703–1729. doi:10.1007/s40265-021-01611-0 PMC8501344 34626339

[16] Jallow S, Govender NP. 2021. Ibrexafungerp: a first-in-class oral triterpenoid glucan synthase inhibitor. J Fungi (Basel) 7:7. doi:10.3390/jof7030163 PMC7996284 33668824

[17] Marcos-Zambrano LJ, Gómez-Perosanz M, Escribano P, Bouza E, Guinea J. 2017. The novel oral glucan synthase inhibitor SCY-078 shows in vitro activity against sessile and planktonic Candida spp. J Antimicrob Chemother 72:1969–1976. doi:10.1093/jac/dkx010 28175309

[18] Watanabe N-A, Miyazaki M, Horii T, Sagane K, Tsukahara K, Hata K. 2012. E1210, a new broad-spectrum antifungal, suppresses Candida albicans hyphal growth through inhibition of glycosylphosphatidylinositol biosynthesis. Antimicrob Agents Chemother 56:960–971. doi:10.1128/AAC.00731-11 22143530 PMC3264227

[19] Chandra J, Ghannoum MA. 2018. CD101, a novel echinocandin, possesses potent antibiofilm activity against early and mature Candida albicans biofilms. Antimicrob Agents Chemother 62:e01750-17. doi:10.1128/AAC.01750-17 29133552 PMC5786756

[20] Parahitiyawa NB, Samaranayake YH, Samaranayake LP, Ye J, Tsang PWK, Cheung BPK, Yau JYY, Yeung SKW. 2006. Interspecies variation in Candida biofilm formation studied using the Calgary biofilm device. APMIS 114:298–306. doi:10.1111/j.1600-0463.2006.apm_394.x 16689830

[21] Coenye T. 2011. Prevention of Candida albicans biofilm formation. Open Mycol J 5:9–20. doi:10.2174/1874437001105010009

[22] Ceri H, Olson ME, Stremick C, Read RR, Morck D, Buret A. 1999. The Calgary biofilm device: new technology for rapid determination of antibiotic susceptibilities of bacterial biofilms. J Clin Microbiol 37:1771–1776. doi:10.1128/JCM.37.6.1771-1776.1999 10325322 PMC84946

[23] Harrison JJ, Ceri H, Yerly J, Stremick CA, Hu Y, Martinuzzi R, Turner RJ. 2006. The use of microscopy and three-dimensional visualization to evaluate the structure of microbial biofilms cultivated in the Calgary Biofilm Device. Biol Proced Online 8:194–215. doi:10.1251/bpo127 17242736 PMC1779619

[24] Jabra-Rizk MA, Falkler WA, Meiller TF. 2004. Fungal biofilms and drug resistance. Emerg Infect Dis 10:14–19. doi:10.3201/eid1001.030119 15078591 PMC3031105

[25] Donlan RM. 2001. Biofilms and device-associated infections. Emerg Infect Dis 7:277–281. doi:10.3201/eid0702.010226 11294723 PMC2631701

[26] Sherry L, Ramage G, Kean R, Borman A, Johnson EM, Richardson MD, Rautemaa-Richardson R. 2017. Biofilm-forming capability of highly virulent, multidrug-resistant Candida auris. Emerg Infect Dis 23:328–331. doi:10.3201/eid2302.161320 28098553 PMC5324806

[27] Verstrepen KJ, Klis FM. 2006. Flocculation, adhesion and biofilm formation in yeasts. Mol Microbiol 60:5–15. doi:10.1111/j.1365-2958.2006.05072.x 16556216

[28] Brandt P, Mirhakkak MH, Wagner L, Driesch D, Möslinger A, Fänder P, Schäuble S, Panagiotou G, Vylkova S. 2023. High-throughput profiling of Candida auris isolates reveals clade-specific metabolic differences. Microbiol Spectr 11:e0049823. doi:10.1128/spectrum.00498-23 37097196 PMC10269459

[29] Muñoz JF, Welsh RM, Shea T, Batra D, Gade L, Howard D, Rowe LA, Meis JF, Litvintseva AP, Cuomo CA. 2021. Clade-specific chromosomal rearrangements and loss of subtelomeric adhesins in Candida auris. Genetics 218:iyab029. doi:10.1093/genetics/iyab029 33769478 PMC8128392

[30] Hirayama T, Miyazaki T, Tanaka R, Kitahori N, Yoshida M, Takeda K, Ide S, Iwanaga N, Tashiro M, Takazono T, Izumikawa K, Yanagihara K, Makimura K, Tsukamoto K, Mukae H. 2025. TAC1b mutation in Candida auris decreases manogepix susceptibility owing to increased CDR1 expression. Antimicrob Agents Chemother 69:e0150824. doi:10.1128/aac.01508-24 39692503 PMC11823642

[31] Chatzimoschou A, Giampani A, Meis JF, Roilides E. 2021. Activities of nine antifungal agents against Candida auris biofilms. Mycoses 64:381–384. doi:10.1111/myc.13223 33270284

[32] Melo AS, Bizerra FC, Freymüller E, Arthington-Skaggs BA, Colombo AL. 2011. Biofilm production and evaluation of antifungal susceptibility amongst clinical Candida spp. isolates, including strains of the Candida parapsilosis complex. Med Mycol 49:253–262. doi:10.3109/13693786.2010.530032 21039308

[33] Kuhn DM, George T, Chandra J, Mukherjee PK, Ghannoum MA. 2002. Antifungal susceptibility of Candida biofilms: unique efficacy of amphotericin B lipid formulations and echinocandins. Antimicrob Agents Chemother 46:1773–1780. doi:10.1128/AAC.46.6.1773-1780.2002 12019089 PMC127206

[34] Larkin E, Hager C, Chandra J, Mukherjee PK, Retuerto M, Salem I, Long L, Isham N, Kovanda L, Borroto-Esoda K, Wring S, Angulo D, Ghannoum M. 2017. The emerging pathogen Candida auris: growth phenotype, virulence factors, activity of antifungals, and effect of SCY-078, a novel glucan synthesis inhibitor, on growth morphology and biofilm formation. Antimicrob Agents Chemother 61:e02396-16. doi:10.1128/AAC.02396-16 28223375 PMC5404565

[35] Ceballos-Garzon A, Monteoliva L, Gil C, Alvarez-Moreno C, Vega-Vela NE, Engelthaler DM, Bowers J, Le Pape P, Parra-Giraldo CM. 2022. Genotypic, proteomic, and phenotypic approaches to decipher the response to caspofungin and calcineurin inhibitors in clinical isolates of echinocandin-resistant Candida glabrata. J Antimicrob Chemother 77:585–597. doi:10.1093/jac/dkab454 34893830 PMC8865013

[36] Ceballos Garzon A, Amado D, Robert E, Parra Giraldo CM, Le Pape P. 2020. Impact of calmodulin inhibition by fluphenazine on susceptibility, biofilm formation and pathogenicity of caspofungin-resistant Candida glabrata. J Antimicrob Chemother 75:1187–1193. doi:10.1093/jac/dkz565 32011702

[37] Simitsopoulou M, Kyrpitzi D, Velegraki A, Walsh TJ, Roilides E. 2014. Caspofungin at catheter lock concentrations eradicates mature biofilms of Candida lusitaniae and Candida guilliermondii. Antimicrob Agents Chemother 58:4953–4956. doi:10.1128/AAC.03117-14 24890585 PMC4136003

[38] Chatzimoschou A, Katragkou A, Simitsopoulou M, Antachopoulos C, Georgiadou E, Walsh TJ, Roilides E. 2011. Activities of triazole-echinocandin combinations against Candida species in biofilms and as planktonic cells. Antimicrob Agents Chemother 55:1968–1974. doi:10.1128/AAC.00959-10 21343465 PMC3088240

[39] Marcos-Zambrano LJ, Gómez-Perosanz M, Escribano P, Zaragoza CO, Bouza E, Guinea J. 2016. Biofilm production and antibiofilm activity of echinocandins and liposomal amphotericin B in echinocandin-resistant yeast species. Antimicrob Agents Chemother 60:3579–3586. doi:10.1128/AAC.03065-15 27021323 PMC4879372

[40] Kovács R, Bozó A, Gesztelyi R, Domán M, Kardos G, Nagy F, Tóth Z, Majoros L. 2016. Effect of caspofungin and micafungin in combination with farnesol against Candida parapsilosis biofilms. Int J Antimicrob Agents 47:304–310. doi:10.1016/j.ijantimicag.2016.01.007 26968084

[41] Uppuluri P, Chaturvedi AK, Srinivasan A, Banerjee M, Ramasubramaniam AK, Köhler JR, Kadosh D, Lopez-Ribot JL. 2010. Dispersion as an important step in the Candida albicans biofilm developmental cycle. PLoS Pathog 6:e1000828. doi:10.1371/journal.ppat.1000828 20360962 PMC2847914

[42] Tits J, Cammue BPA, Thevissen K. 2020. Combination therapy to treat fungal biofilm‐based infections. Int J Mol Sci 21:8873. doi:10.3390/ijms21228873 33238622 PMC7700406

[43] Anon, Clinical and Laboratory Standards Institute. 2017. M27. Reference method for broth dilution antifungal susceptibility testing of yeasts. 4th ed. CLSI, Wayne, PA, USA.

[44] CLSI. 2022. CLSI supplement M27M44S. Performance standards for antifungal susceptibility testing of yeast. 3rd ed. Clinical and Laboratory standars institute.

[45] Ceballos-Garzon A, Holzapfel M, Welsch J, Mercer D. 2025. Identification and antifungal susceptibility patterns of reference yeast strains to novel and conventional agents: a comparative study using CLSI, EUCAST and Sensititre YeastOne methods. JAC Antimicrob Resist 7:dlaf040. doi:10.1093/jacamr/dlaf040 40110552 PMC11920621

[46] Ceballos-Garzon A, Roman E, Pla J, Pagniez F, Amado D, Alméciga-Díaz CJ, Le Pape P, Parra-Giraldo CM. 2022. CRISPR-Cas9 approach confirms calcineurin-responsive zinc finger 1 (Crz1) transcription factor as a promising therapeutic target in echinocandin-resistant Candida glabrata. PLoS One 17:e0265777. doi:10.1371/journal.pone.0265777 35303047 PMC8932611

[47] Dudek B, Brożyna M, Karoluk M, Frankiewicz M, Migdał P, Szustakiewicz K, Matys T, Wiater A, Junka A. 2024. In vitro and in vivo translational insights into the intraoperative use of antiseptics and lavage solutions against microorganisms causing orthopedic infections. Int J Mol Sci 25:12720. doi:10.3390/ijms252312720 39684431 PMC11641374

[48] Ravi NS, Aslam RF, Veeraraghavan B. 2019. A new method for determination of minimum biofilm eradication concentration for accurate antimicrobial therapy. Methods Mol Biol 1946:61–67. doi:10.1007/978-1-4939-9118-1_6 30798544

[49] Repp KK, Menor SA, Pettit RK. 2007. Microplate Alamar blue assay for susceptibility testing of Candida albicans biofilms. Med Mycol 45:603–607. doi:10.1080/13693780701581458 17885957

[50] Gómez-Casanova N, Lozano-Cruz T, Soliveri J, Gomez R, Ortega P, Copa-Patiño JL, Heredero-Bermejo I. 2021. Eradication of Candida albicans biofilm viability: in vitro combination therapy of cationic carbosilane dendrons derived from 4-phenylbutyric acid with AgNO_3_ and EDTA. J Fungi (Basel) 7:574. doi:10.3390/jof7070574 34356953 PMC8305162

