# Supplementary material for: Antibiofilm activity of manogepix, ibrexafungerp, amphotericin B, rezafungin, and caspofungin against Candida spp. biofilms of reference and clinical strains
Source: Antimicrob Agents Chemother. 2025 May 15;69(6):e00137-25. doi: 10.1128/aac.00137-25 (PMC12135511; doi:10.1128/aac.00137-25)
Supplement: Fig. S1 — Biofilm formation capacity of Candida albicans, Candida auris, and Candida parapsilosis assessed using the Calgary Biofilm Device. [file aac.00137-25-s0001.pdf]

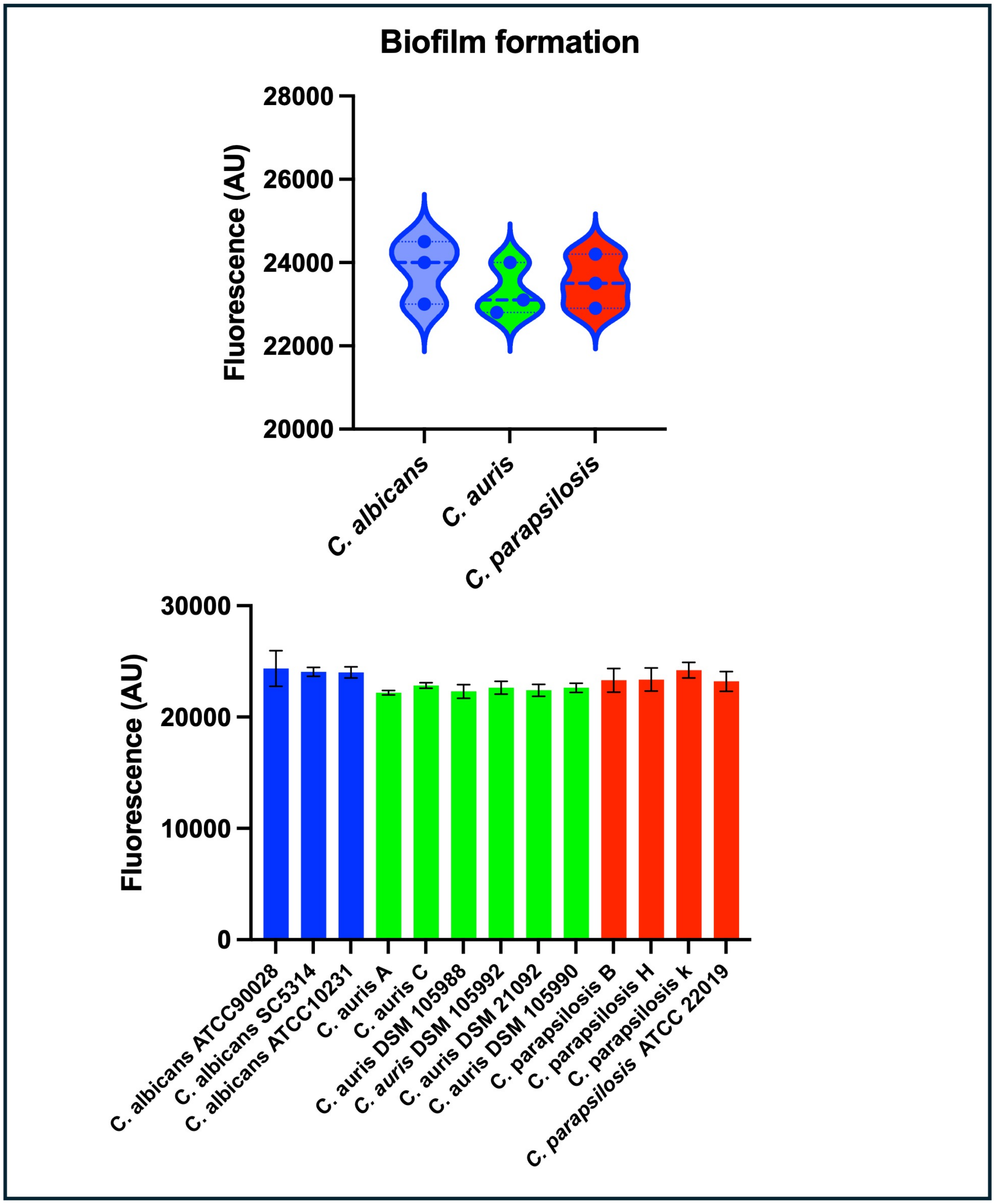

Figure S1. Biofilm formation capacity of Candida albicans, Candida auris, and Candida parapsilosis assessed using the Calgary Biofilm Device. On top biofilm formation by species, showing mean fluorescence intensity. At the bottom biofilm formation by strains. Data are expressed as arbitrary fluorescence units (AU).
